# Uncovering the Harms of Treating Clostridioides difficile Colonization

**DOI:** 10.1128/mSphere.01296-20

**Published:** 2021-01-13

**Authors:** Christopher R. Polage, Nicholas A. Turner

**Affiliations:** a Duke University Health System, Clinical Microbiology Laboratory, Durham, North Carolina, USA; b Duke University School of Medicine, Department of Pathology, Durham, North Carolina, USA; c Duke University School of Medicine, Division of Infectious Diseases, Durham, North Carolina, USA

**Keywords:** *Clostridioides difficile*, clinical trials, vancomycin

## Abstract

Patients with toxin-negative Clostridioides difficile-positive diarrhea are often treated with oral vancomycin with the assumption that treatment is more beneficial than harmful. However, this hypothesis has never been formally tested, and recent studies suggest that most such patients recover quickly without treatment and can be colonized rather than infected. Fishbein et al. conducted a prospective, placebo-controlled randomized trial to systematically evaluate the effects, risks, and benefits of oral vancomycin in these patients (S. R. S. Fishbein, T. Hink, K. A. Reske, C. Cass, et al., mSphere 6:e00936-20, 2020, https://doi.org/10.1128/mSphere.00936-20). Although small, the results are intriguing and suggest the adverse antibiotic-induced effects of vancomycin outweigh the clinical benefit when colonization is more likely than disease.

## Abstract (precis)

Patients with toxin-negative Clostridioides difficile-positive diarrhea are often treated with oral vancomycin with the assumption that treatment is more beneficial than harmful. However, this hypothesis has never been formally tested, and recent studies suggest that most such patients recover quickly without treatment and can be colonized rather than infected.

## COMMENTARY

Ten years ago, Clostridioides difficile was assumed to always be pathogenic in patients with diarrhea; treatment with anti-C. difficile antibiotics was recommended whenever C. difficile was detected, with the belief that antibiotics were needed and not harmful (1). This dogma was questioned by studies showing that fecal toxin status had clinical prognostic significance, and patients usually recovered with minimal or no antibiotic treatment when free fecal toxins were not detected (2, 3). Updated clinical guidelines acknowledged the possibility that some patients with C. difficile*-*associated diarrhea were colonized rather than infected and made treatment optional when fecal toxins were not detected (4, 5). However, many providers continue to prescribe antibiotics routinely for patients with toxin-negative C. difficile-associated diarrhea for a variety of reasons (e.g., toxin test not performed or the belief that antibiotic treatment is more beneficial than harmful overall).

On this backdrop, Fishbein et al. conducted a blinded, randomized controlled trial of vancomycin versus placebo to better define the benefits and risks of antibiotic treatment in patients with fecal toxin-negative C. difficile-positive diarrhea (6). Although small, the study is remarkable for its robust design and comprehensive assessment of treatment implications in a population where the need for treatment is often unclear and the harms of treatment are unseen (6). Beyond clinical outcomes, the authors analyzed the effects of treatment on gut microbiome diversity as well as presence, shedding, and environmental contamination with C. difficile and other antibiotic-resistant organisms (AROs).

Performing these additional analyses was wise, because antibiotic-induced dysbiosis plays a central role in the colonization, pathogenesis, and transmission of C. difficile and other gut-colonizing AROs in health care facilities (7, 8). Antibiotic-induced reductions in commensal bacteria and gut metabolome alterations facilitate C. difficile germination, growth, toxin production, and disease (9). Other antibiotic-induced gut microbiota and immune changes facilitate vancomycin-resistant *Enterococcus* (VRE) colonization, overgrowth, and infection (10). Moreover, efforts to protect the gut microbiota (through antibiotic stewardship practices) or restore commensal diversity (through fecal microbiota transplantation) are emerging strategies to prevent C. difficile and ARO colonization, infection, and transmission.

There are several interesting and important takeaways from this study. First, although the study was not intended or powered to prove the safety of withholding antibiotics from patients with fecal toxin-negative C. difficile-positive diarrhea, it is worth noting that treating these patients with vancomycin was not associated with an obvious clinical benefit. Only a single patient from the placebo group transitioned to toxin-positive status during the study period, and there was no gross difference in the duration of diarrhea between groups.

Second, oral vancomycin can have strongly damaging effects on the microbiome. While fluoroquinolones, advanced-generation cephalosporins, and lincosamides receive the most attention, nearly any antibiotic can injure or alter the microbiome. Prior high-throughput sequencing efforts have shown dramatic reductions in gut microbial diversity following the receipt of oral vancomycin, sometimes lasting months (11–13). Vancomycin administration was also associated with significant changes in the gut microbiota community structure in this study, reminding us that vancomycin is not benign. Future studies should compare the effects of different doses of vancomycin to help us understand if prophylaxis doses have a similar or lesser effect on the gut microbiome.

Third, treatment with oral vancomycin fails to reduce C. difficile colonization or shedding relative to placebo overall. This finding aligns with our current understanding of C. difficile colonization; while antibiotics used to treat C. difficile are generally active against vegetative cells and can inhibit germination of spores, none are capable of destroying spores (14). Between the inability to eliminate C. difficile spores and collateral damage to the microbiome during treatment, vancomycin may actually leave recipients at increased risk of C. difficile colonization after treatment (15). Fishbein et al.’s observation of ongoing C. difficile shedding regardless of vancomycin treatment validates these concerns.

Fourth, vancomycin may increase the risk of colonization and/or subsequent infection with other AROs. Although underpowered to detect a difference in VRE colonization, Fishbein et al. found an increase in E. faecium abundance in patients randomized to vancomycin treatment for C. difficile. The validity of this observation is supported by prior data, including a meta-analysis linking vancomycin receipt with a dramatically increased risk for VRE colonization (16).

Collectively, vancomycin is at best a double-edged sword. Although entirely appropriate for the treatment of true C. difficile colitis, the risk-benefit balance seems much less favorable for colonized and toxin-negative patients with nonsevere diarrhea, as in this study. Although some clinicians have advocated vancomycin for the prophylactic treatment of C. difficile in colonized individuals receiving additional antibiotics, this study by Fishbein et al. highlights the need for caution. Despite intuitive appeal, the prophylactic use of vancomycin has had mixed results with respect to clinical outcomes in the few existing retrospective studies to date (17–19). Besides having limited clinical benefit, Fishbein et al. remind us of the harms of vancomycin treatment, including damage to the microbiome, potential for promoting prolonged C. difficile shedding, and the potential for increasing the risk of colonization with other AROs. In short, antibiotic treatment does not appear to be the answer for C. difficile*-*colonized patients and should probably be used more judiciously in toxin-negative patients with diarrhea when there is no evidence of severe or fulminant disease. The most recent guidance from the European Society of Clinical Microbiology and Infectious Diseases (ESCMID) recommends the use of multistep testing with a toxin assay and clinical evaluation of toxin-negative patients before treatment (4). The Infectious Diseases Society of America (IDSA) recommends testing and treatment only for patients with clinically significant diarrhea and includes the option of multistep testing with a toxin assay to help inform treatment decisions (5).

More research is needed to expand our understanding of how various clinical antibiotics affect the microbiome and resistance or susceptibility to colonization by C. difficile and other AROs. Perhaps injury to the microbiome might even be incorporated among the adverse effects assessed during drug development. Better yet, given the emerging success of microbial reconstitution strategies in managing recurrent C. difficile, efforts to protect microbiome integrity and diversity seem to have significant potential for preventing colonized patients from progressing to disease (20). Novel strategies such as microbial reconstitution therapy to replenish damaged gut microbiota or nonabsorbable beta-lactamases to reduce injury to gut microbiota in the first instance may hold promise for the future (21). At least with respect to C. difficile, perhaps it is time to shift the focus from always treating the pathogen to promoting and restoring host resistance to infection.
